# Supplementary material for: Development of Loop-Mediated Isothermal Amplification Assay for Rapid Detection of Cucurbit Leaf Crumple Virus
Source: Int J Mol Sci. 2020 Mar 4;21(5):1756. doi: 10.3390/ijms21051756 (PMC7084362; doi:10.3390/ijms21051756)
Supplement: Supplementary file 1 [file ijms-21-01756-s001.pdf]

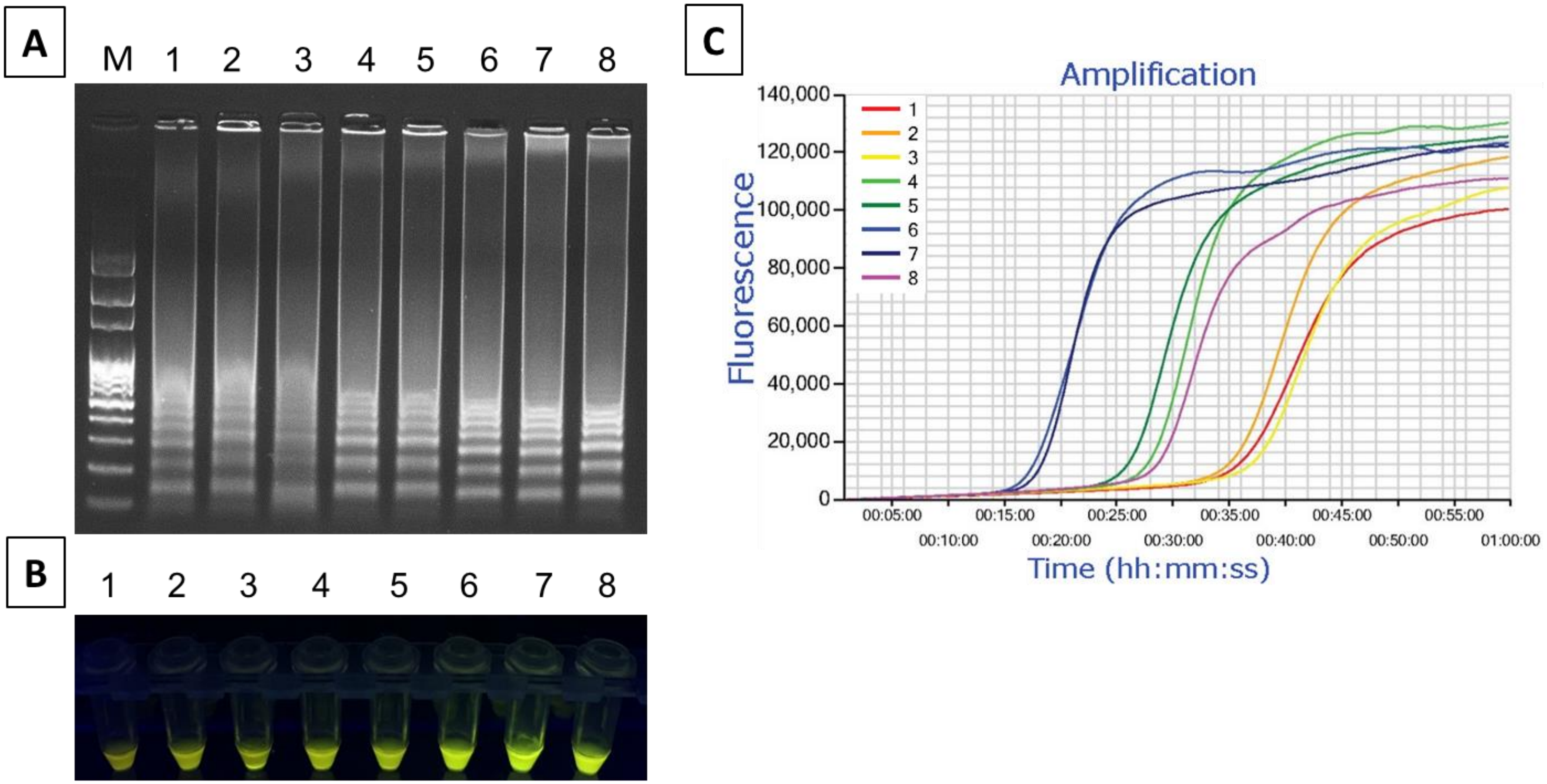

Supplementary Figure 1

Optimization of temperature of LAMP reaction for CuLCrV. Effect of temperature from 66 to 73°C where No. 1, 2, 3, 4, 5, 6, 7 and 8 is 66, 67, 68, 69, 70, 71, 72 and 73°C, respectively. Results were analyzed by agarose gel electrophoresis (A), SYBR™ green 1 DNA gel staining for visual amplification (B) and real-time amplification by Genie® III (C). Lane M: 100 bp DNA ladder.

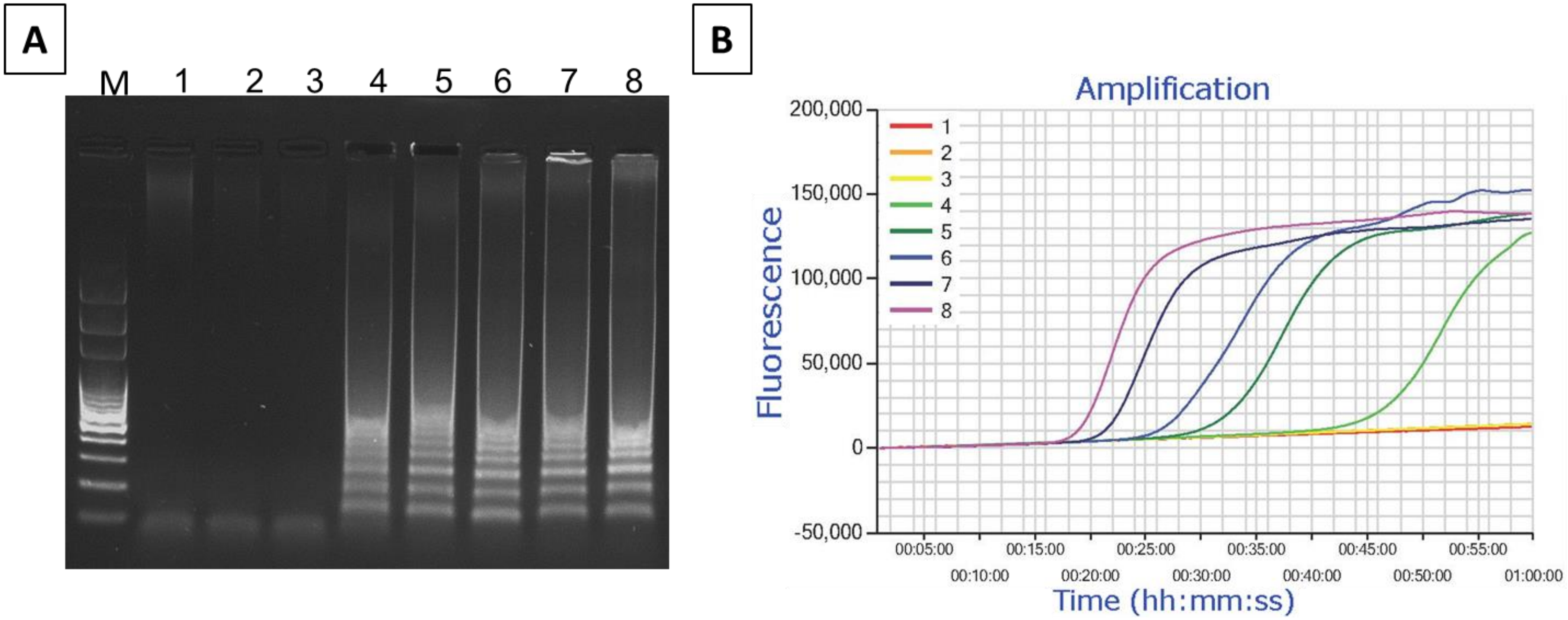

Supplementary Figure 2

Failure of LAMP amplification for CuLCrV below temperature of 65°C using LavaLAMP™ DNA master mix. Effect of temperature from 62 to 69°C where No. 1, 2, 3, 4, 5, 6, 7 and 8 is 62, 63, 64, 65, 66, 67, 68 and 69°C, respectively. Results were analyzed by agarose gel electrophoresis (A), and real-time amplification by Genie® III (B). Lane M: 100 bp DNA ladder.

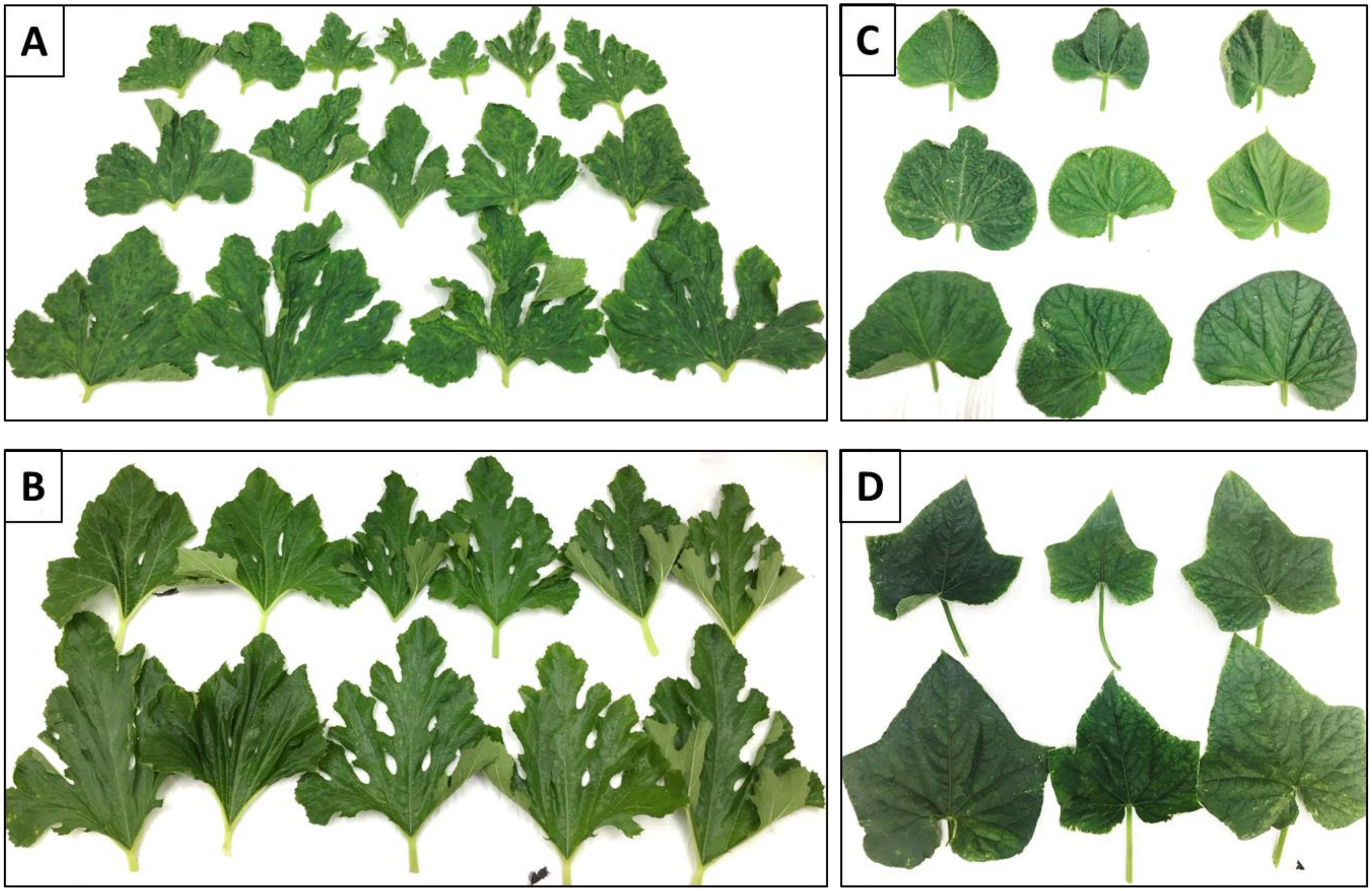

Supplementary Figure 3

Photographs of CuLCrV infected symptomatic (A, C) and asymptomatic (B, D) winter squash (A, B) and cucumber (C, D) leaf samples used in this study. The leaves from the upper panels (A, C) have typical yellow chlorotic spots, interveinal yellowing, mosaic, and leaf curling and crumpling symptoms, while the leaves from the lower rower panels (B, D) show no symptoms.

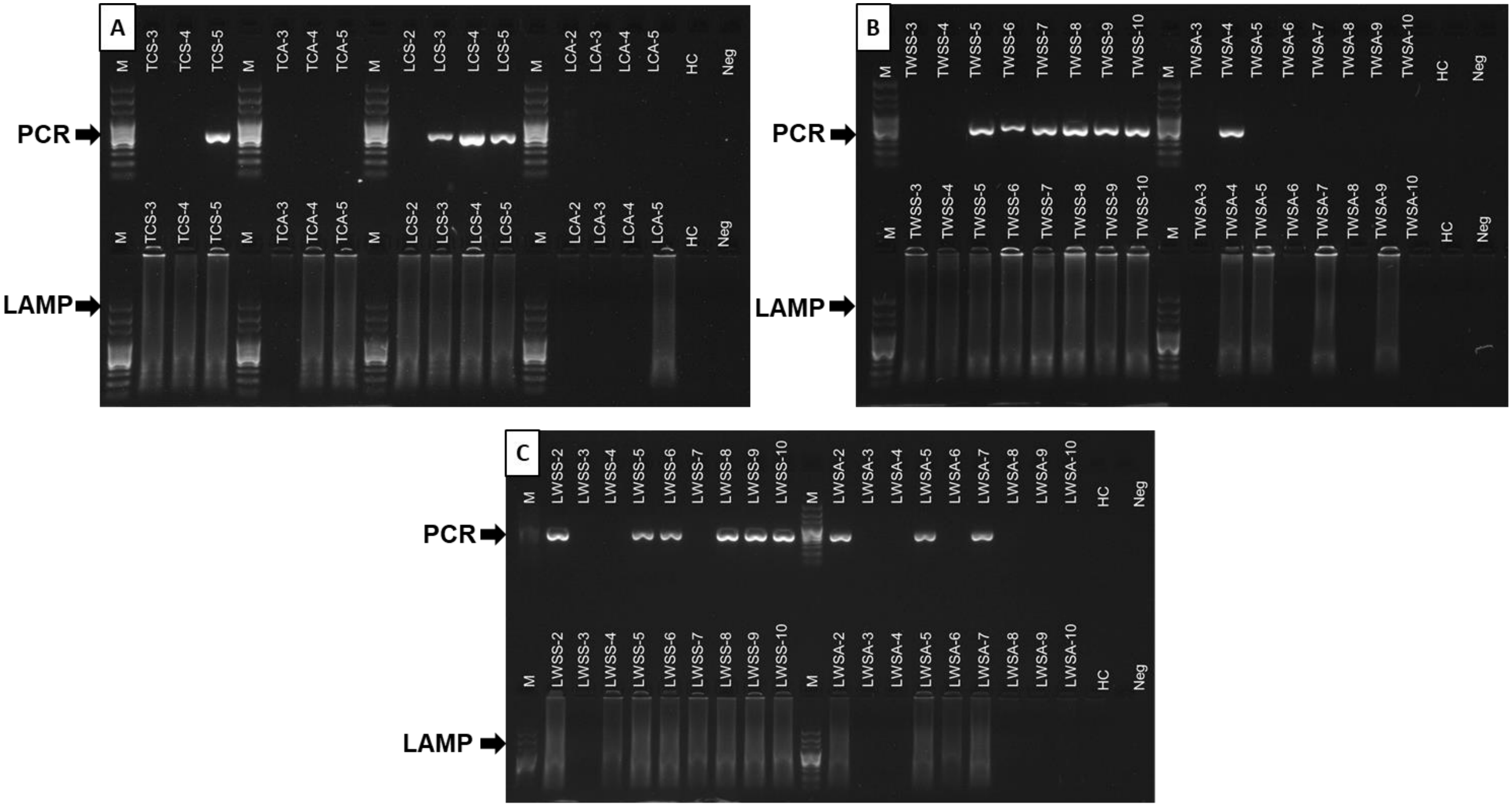

Supplementary Figure 4

Detection of CuLCrV from symptomatic and asymptomatic cucumber and winter squash leaf samples collected from Tift and Lowndes county by LAMP and PCR visualized by agarose gel electrophoresis. Here, TCS: Tift county cucumber symptomatic leaf samples, TCA: Tift county cucumber asymptomatic leaf samples, LCS: Lowndes county cucumber symptomatic leaf samples, LCA: Lowndes county cucumber asymptomatic leaf samples, TWSS: Tift county winter squash symptomatic leaf samples, TWSA: Tift county winter squash asymptomatic leaf samples, LWSS: Lowndes county winter squash symptomatic leaf samples, LWSA: Lowndes county winter squash asymptomatic leaf samples, HC: healthy control, Neg: negative control, M: 100 bp ladder marker.

Supplementary Table 1. Oligonucleotide sequences used for PCR, real-time PCR and LAMP in this study.

Assay	Primers	Sequence (5′-3′)	Reaction condition	Product size (bp)	Reference
PCR	CuLCrV-F<br>CuLCrV-R	ATATCATGATTTTCGAGTACATAG<br>AATGAAAGCCTAAGAGAGTG	94°C, 3 min; 35 × (94°C, 30 s; 54°C, 45 s; 72°C, 1 min); 72°C, 7min	541	This study
	SLCV-F<br>SLCV-R	TTTATGTGGGCCCTCGACC<br>AGGACGACGGTCTTGAACC	94°C, 3 min; 35 × (94°C, 30 s; 58°C, 30 s; 72°C, 30 min); 72°C, 7min	612	[5]
	TYLCV-F<br>TYLCV-R	ACG CAT GCC TCT AAT CCA GTG TA<br>CCA ATA AGG CGT AAG CGT GTA GAC	94°C, 3 min; 35 × (94°C, 30 s; 56°C, 30 s; 72°C, 45s); 72°C, 7min	543	[9]
	SMLCV-F<br>SMLCV-R	GGAGAGCATCACTGTGTATCAG<br>CCCAGGAAACAGCTCTAACAA	94°C, 3 min; 35 × (94°C, 30 s; 56°C, 30 s; 72°C, 30s); 72°C, 7min	317	This study
	CYSDV-CP-F<br>CYSDV-CP-R	ATGGCGAGTTCGAGTGAGAATAA<br>ATTACCACAGCCACCTGGTGCTA	94°C, 3 min; 35 × (94°C, 30 s; 50°C, 45 s; 72°C, 1 min); 72°C, 7min	755	[8]
	SqVYV-CP-F<br>SqVYV-CP-R	CCCTCGGAGAACTTG ATATGGAAGCAC<br>CGCGTC CTTCCTCTCCAGGCGCTG	94°C, 3 min; 35 × (94°C, 30 s; 63°C, 1 min; 72°C, 1 min); 72°C, 7min	1071	[1]
	CCYV-HSP-F<br>CCYV-HSP-R	TGCGTATGTCAATGGTGTTATG<br>ATCCTTCGCAGTGAAAAACC	94°C, 3 min; 35 × (94°C, 30 s; 55°C, 30s; 72°C, 30s); 72°C, 7min	462	[4]
	ToCV-CP-F<br>ToCV-CP-R	ATGGAGAACAGTGCTGTTGC<br>TTAGCAACC AGTTATCGATGC	94°C, 3 min; 35 × (94°C, 30 s; 57°C, 45s; 72°C, 45s); 72°C, 7min	774	[3]
qPCR	CuLCrVq-F<br>CuLCrVq-R	TGTGCATATCTGACGTGACCC<br>TTCGTCCATCCAGATCTTCCC	95°C, 120 s (optics off); 40 × (95°C,10 s (optics off);<br>60°C, 50 s (optics on)); 60°C-95°C, 0.2°C/s	104	This study
	SLCV-F<br>SLCV-R	TTTATGTGGGCCCTCGACC<br>AGGACGACGGTCTTGAACC		191	[5]
	TYLCV-OM-F<br>TYLCV-OM-R	GAAGCCCTGATGTTCCCCGTGG<br>GATTTAACACAGAACCTCTTACC		159	[2]
	SMLCVq-F<br>SMLCVq-R	GAGCTTCTGGGACGACTTATTT<br>CCCAGGAAACAGCTCTAACAA		108	This study
	TSWV-N-F<br>TSWV-N-R	GCTTCCCACCCTTTGATTC<br>ATAGCCAAGACAACACTGATC		140	[6]
LAMP	CuLCrV-F3<br>CuLCrV-B3<br>CuLCrV-FIP<br>CuLCrV-BIP<br>CuLCrV-LF<br>CuLCrV-LB	AAATCCAGGTGACCGCTAAG<br>CCTGAAACGTGGGCATGTC<br>CGGCCAATGCGAGCTAGAAGTAGCCTAACGAACGGGACCA<br>AGCGGCATCCCTCCCAAAATCATCGTTCACTCTCGTTCCA<br>TCAAGAACGGGGAAGACGA<br>AATCATCAGCCCACTCCTGT	90°C, 3 min; 71°C, 60 min; 98-80°C, 0.05°C/s	NA	This study
Rubisco	Rbc-F<br>Rbc-R	TACTTGAACGCTACTGCAG<br>CTGCATGCATTGCACGGTG	94°C, 2 min; 35 × (94°C, 30 s; 50°C, 30s; 68°C, 45s); 68°C, 5 min	186	[7]

References for primers:

1. Adkins, S.; Webb, S.E.; Baker, C.A.; Kousik, C.S. *Squash vein yellowing virus* detection using nested polymerase chain reaction demonstrates that the cucurbit weed *Momordica charantia* is a reservoir host. *Plant disease* ***2008, 92, 1119-1123.***
2. Ammara, U.; Al-Sadi, A.M.; Al-Shihi, A.; Amin, I. Real-time qPCR assay for the TYLCV titer in relation to symptoms-based disease severity scales. *International Journal of Agriculture and Biology* ***2017, 19, 145-151.***
3. Çevik, B.; Kıvrak, H.; Şahin-Çevik, M. Development of a graft inoculation method and a real-time RT-PCR assay for monitoring *Tomato chlorosis virus* infection in tomato. *Journal of virological methods* ***2019, 265, 1-8.***
4. Gyoutoku, Y.; Okazaki, S.; Furuta, A.; Etoh, T.; Mizobe, M.; Kuno, K.; Hayashida, S.; Okuda, M. Chlorotic yellows disease of melon caused by *Cucurbit chlorotic yellows virus*, a new crinivirus. *Japanese Journal of Phytopathology (Japan)* ***2009.***
5. Kuan, C.-P.; Wu, M.-T.; Lu, Y.-L.; Huang, H.-C. Rapid detection of *Squash leaf curl virus* by loop-mediated isothermal amplification. *Journal of virological methods* ***2010, 169, 61-65.***
6. Okazaki, S.; Okuda, M.; Komi, K.; Yamasaki, S.; Okuda, S.; Sakurai, T.; Iwanami, T. The effect of virus titer on acquisition efficiency of *Tomato spotted wilt virus* by *Frankliniella occidentalis* and the effect of temperature on detectable period of the virus in dead bodies. *Australasian Plant Pathology* ***2011, 40, 120-125.***
7. Sanchez-Navarro, J.; Aparicio, F.; Herranz, M.; Minafra, A.; Myrta, A.; Pallas, V. Simultaneous detection and identification of eight stone fruit viruses by one-step RT-PCR. *European Journal of Plant Pathology* ***2005, 111, 77-84.***
8. Steel, E.; Barker, I.; Danks, C.; Coates, D.; Boonham, N. A. Tumefaciens-mediated transient expression as a tool for antigen production for *Cucurbit yellow stunting disorder virus*. *Journal of virological methods* ***2010, 163, 222-228.***
9. Zhang, B.; Zou, C.; Hu, Q. Effects of *Isaria fumosorosea* on TYLCV (tomato yellow leaf curl virus) accumulation and transmitting capacity of *Bemisia tabaci*. *PloS one* ***2016, 11.***

Supplementary Table 2. Summary statistics for the overall match between CuLCrV and 5 other closely related begomoviruses for LAMP primer binding sites[1]

Name of Viruses	Identity (%)	Protein Identity (%)	Presence of SNPs (Y/N)	No. of SNPs (bp)	Presence of In/Del mutation (Y/N)	No. of In/Del mutation (bp)
BCMV	79.8	84.1	Yes	39	No	N/A
MCLCV	79.8	78.3	Yes	52	Yes (Insertion)	30
SYMMV	79.7	77.4	Yes	47	Yes (Insertion)	30
SLCV	81.8	83.6	Yes	46	Yes (Deletion)	6
SMLCV	83.2	86	Yes	41	No	N/A

[1] Identity % = Pairwise identity between nucleotide sequences of CuLCrV DNA-A and other closely related begomovirus DNA-A; Protein Identity (%) = Pairwise identity between amino acid sequences of CuLCrV DNA-A and other closely related begomovirus DNA-A; Presence of SNPs (Y/N) = whether there is any single nucleotide polymorphism (SNP) present within the above stated begomoviruses for CuLCrV-LAMP primers binding sites; No. of SNPs (bp) = Number of SNPs present within the above listed begomoviruses for CuLCrV-LAMP primers binding sites; Presence of In/Del mutation (Y/N) = whether there is any insertion or deletion mutation present within the above stated begomoviruses for CuLCrV-LAMP primers binding sites; No. of In/Del mutation (bp) = Number of insertion or deletion mutation present within the above mentioned begomoviruses for CuLCrV-LAMP primers binding sites. N/A = Not applicable, bp = base pair.

Supplementary Table 3. Optimization of temperature of the LAMP reaction for CuLCrV using real-time LAMP detection system Genie® III. Selected temperature for LAMP amplification is highlighted with a bold format.

Tm_{amp} (°C)[a]	Ti_{amp} (Min:S)[b]	Ta (°C)[c]
65	49.3	86.56
66	38.3	86.53
67	38	85.94
68	39.15	84.68
69	29.15	85.22
70	27.3	85.95
**71**	**18.15**	**86.58**
72	19.3	86.29
73	30.15	86.53

[a] Amplification temperature in degree Celsius (°C)
[b] Amplification time in minutes and seconds (Min:S)
[c] Annealing temperature in degree Celsius (°C)
